# Combination of Cohesive and Non-Cohesive Gold in Filling

**Published:** 1894-06

**Authors:** C. N. Johnson

**Affiliations:** Chicago, Ill.


Article ix.

### COMBINATION OF COHESIVE AND NON-COHESIVE GOLD IN FILLING

C. N. JOHNSON, L. D. S, D. D. S., CHICAGO, ILL.

In the use of the terms cohesive and non co-hesive gold, many writers seem to assume that there is no gradation in character between a gold which cannot be made to weld with any amount of pressure—non-cohesive, and one which welds on the very slightest provocation to a degree which renders it harsh and unyielding in its working qualities. To them the term cohesive gold evidently signifies a substance of a sticky, unmanageable nature, a pellet of which when accidently dropped in a wrong position in the process of filling, is irretrievable fastened to this false situation, and cannot be shifted or worked into a more desirable position on account of its persistent tenacity in cohering to the surface of the condensed gold already in the cavity. Gold having this extreme cohesiveness is a rarity. If the cohesive gold in universal use by the profession possessed such remarkable cohesive properties as this, we we should see very many more leaky fillings than we do, and we should also see the surfaces of gold fillings retain a polish better than most of them do. The truth probably is, that among all the golds on the market to-day there is comparatively little that is absolutely non-cohesive, unless made so through special preparation by the dentist himself, and a smaller per cent still that could be claimed as perfectly cohesive, unless other means than the ordinary methods of annealing are resorted to. It may therefore be assumed that most of the gold inserted in teeth at the present time is used in a semi-cohesive condition, and this condition is more favorable to good results than would an equal use of either non-cohesive or a thoroughly cohesive gold.

But there are places where we need a non- cohesive gold, and others where the gold should be as cohesive as we can make it and it is to the definite arrangement of the different forms, non- cohesive, semi-cohesive and cohesive in the same cavity that your attention is invited.

Let us suppose a cavity properly prepared on the proximate surface of a bicuspid, extending well over on the occlusal surface. The proper preparation of a cavity like this implies that the cervical outline shall be as nearly horizontal as circumstances will permit. An abrupt angle between the cervical and lateral outlines is of course impracticable, but the curve should be rather a sharp one, to give a broad, level base to the cavity.

The non-cohesive gold to be used should be in the form of cylinders, and the cylinders should be sufficiently large so that when laid along the floor of the cavity with the ends facing the buccal and lingual walls, and there compressed, the layers of foil forming the cylinder will be wide enough to extend from the wall of the cavity nearest the pulp to the cervical border, and overlap it.

The portion of the cavity in which the fiilling should be started in these cases, is usually in the angle formed by the junction of the lingual wit the cervical wall, or, in other words, in in the cervico-lingual corner. This statement is made with the reservation that the method of procedure must vary. We cannot always obtain an ideally formed cavity, and any modification in the form of the cav- may modify the starting point of the filling as other processes of operating are modified by circumstances. A large cylinder of non-cohesive gold is lain on its side in the cervico-lingual corner, with one end looking toward the buccal wall and the other slightly turned up toward the occlusal surface. This is carefully "coaxed" into position without breaking up the layers forming the cylinder or disintegrating the mass. No attempt is made to condense this first cylinder. It is partially compressed to ascertain whether or not there is going to be a sufficient mass of gold which, when condensed, will perfectly fill any undercut and extend across the floor of the cavity and slightly overlap the cervical border. In shallow cavities one cylinder, of a size so large that it will barely pass through the orifice of the cavity without being torn or disintegrated, will sometimes accomplish this, but in many cases it will require two or three cylinders before sufficient bulk of non-cohesive gold is obtained. When the required amount is in position, a smaller cylinder of cohesive gold is annealed as foil, and this is placed on the uncondensed non-cohesive gold. A plugger point, shaped to conform somewhat to the walls of the cavity, is then brought down on the cohesive cylinder, and force by hand pressure is exerted to drive the mass directly toward the cervico-lingual corner. This wedges the gold firmly into the angle of the cavity. The non-cohesive is irresistibly carried before the cohesive, and is adapted to the walls without being punctured and squeezed out of the cavity, as would be the case if the plugger point were brought directly on it. The cohesive cylinder, when thoroughly annealed, forms an impenetrable mat against which the plugger may be pressed with sufficient force to insure adaptation and condensation, without breaking up the mass of gold into fragments. The cohesive gold is carried into the substance of the non-cohesive, and lends solidity and stability to the first portion of the filling The only means by which these first pieces of non-cohesive gold can be condensed independently of the cohesive cylinder without danger of disintegration, is by the use of broad pointed pluggers, and with broad pluggers there is no assurance that adaptation is perfect in undercuts or grooves. The spreading qualities often attributed to non-cohesive gold must not be depended on to any appreciable extent. It is probably safer to assume that gold, whether cohesive or non-cohesive will go only where it is forced to go by definite and direct pressure, than to depend on a lateral moving of the mass when the plugger point is brought to bear directly on it.

When the first pieces of gold are anchored in position it will be found that the cervico-lingual corner of the cavity is well covered over, with the gold extending further along the cervical than the lingual border. It is sometimes necessary to use two or even three cylinders of cohesive gold to overlie and protect the non-cohesive, but no cylinder except the first should be annealed to a high temperature. The object of high annealing in the first cohesive cylinder is to obtain a mass of gold the layers of which will weld on the slightest pressure, so that when the plugger point is brought against it the layers cling together so tenaciously that great force may be exerted without puncturing it. This quality not being so necessary in the succeeding cylinders they are rendered only semicohesive.

When the filling has progressed thus far in the ordinary cavity of a bicuspid, it will usually be found that the gold extends more than half way across the servical wall in the direction of the buccal wall. The conformation of the cavity viewed at this stage of the operation shows a decided depression at the cervico-buccal corner, and into this depression a cylinder of non-cohesive gold is placed, in the same general arrangement as with the first cylinder in the cervico-lingual cornes. The end looking toward the lingual wall will slightly overlap the gold already in place, while the other end will curve toward the occlusal surface along the cervical third of the buccal wall. The same plan of anchorage is followed here as with the other corner, a retaining instrument being held firmly against the condensed portions of gold to keep the mass from moving while the process of wedging between buccal and lingual wall is progressing. When the span is made between these two walls and the gold thoroughly condensed along the whole cervical outline, and up to the cervical third of the lingual and buccal walls, we have a firm foundation for the filling, which, if the cavity has been properly formed, cannot be tipped or dislodged in any way by mallet force during the subsequent process of the operation.

Up to the time when the connection is fully made between the lingual and buccal walls, and the cervical outline well covered, a mallet of any kind is ordinarily contraindicated. Hand pressure vigorously exerted in the proper direction, will insure good adaptation, with less danger of injury to margins. After the floor of the cavity is covered a mallet should be used to complete the condensation. The first blows should be struck midway between the buccal and lingual walls, and the plugger point carried in either direction from the center toward the walls, the last blow on the gold immediately overlying the walls.

The cavity is now filled about one third full. A cushion of non-cohesive gold covers the margins, while solidity is secured by a layer of semi-cohesive gold over it. The filling is slightly higher along the buccal and lingual walls than in the center, while the whole mass is firmly anchored in place. The most difficult part of the operation is completed, and the process from now on consists merely in building back and forth between buccal and lingual walls. This, for the most part; is done with semi-cohesive gold, but at any point in the cavity where there is a decided undercut or groove, a cylinder of non-cohesive gold should be laid into it and driven to place with a smaller cylinder of cohesive gold. As the surface of the filling is being reached, and all margins are protected, the gold should be annealed thoroughly to gain the benefit of its greatest cohesive properties.

When the occlusal portion of the cavity is approached a definite plan of anchorage should be followed to insure firmness of the filling at this point. If the groove running between the cusps and leading to the depression near the opposite marginal ridge is deep, a cylinder of non-cohesive gold should be laid in it with the ends looking mesially and distally. A cohesive cylinder should be placed on this and thoroughly condensed into it, the end of the latter overlapping the cohesive gold already in place. From this point to the completion of the filling, the gold should be made as cohesive as possible. Where the groove is shallow, cohesive gold should be used throughout the entire occlusal portion of the filling. In case the depression near the opposite marginal ridge dips into the dentine much deeper than the groove between the cusps, it should be filled with non-cohesive gold nearly to a level with the floor of the groove before any gold is placed in the groove itself. In these cases the groove is usually shallow, and we now have two sections to the filling—the main body in the proximate cavity, and the smaller piece in the depression on the occlusal surface. A cylinder of cohesive gold is then placed in the groove, with one end over-lapping the distal section and the other the mesial, and condensed so as to b nd the two portions together. Gold thoroughly annealed is used to complete the filling.

It will be seen that the general arrangement of the different forms of gold in a filling of this kind is as follows: Non-cohesive gold lines the cervical portion of the cavity and fills any deep depressions or undercuts, semi-cohesive gold foıms the bulk of the filling, while a decidedly cohesive gold covers the surface.

The selection of plugger points for this method of filling is important. The question of form cannot be treated on in this paper, but a word may be said as to serrations.

For the purpose of fastening the first pieces of cohesive gold into the substance of the non-cohesive, pluggers with deep serrations are called for. Deep serrations secure an interlacing of the layers, and result in a more stable union between the two forms of gold than can be accomplished with shallow serrations. In the process of building up the filling with semi-cohesive gold when we are simply adding piece after piece to the main mass to increase its bulk, and not working around margins, deep serrations are indicated on the same principle. But in condensing over margins deep serrations are dangerous, on account of the tendency to puncture the gold and injure the enamel borders. Shallow serrations should be used along all margins. As the surface of the filling is approached and the gold is rendered more cohesive, the necessity for deep serrations passes away, and the best results are obtained by using shallow serrations. When the last piece of gold is in place, condensation may be completed with pluggers having no serrations.

The statement just made with regard to deep serrations applies only to hand pressure, the hand mallet, or automatic mallet. With the engine, electric, or any of the rapid acting mallets, deep serrations are contraindicated.

In the present paper only one form of filling has been described. A detailed description of the methods to be followed in the different classes of cavities would extend the paper beyond the limits assigned to it.—*Dental Review.*

---

